# α-synuclein interacts with SOD1 and promotes its oligomerization

**DOI:** 10.1186/s13024-015-0062-3

**Published:** 2015-12-08

**Authors:** Anika M. Helferich, Wolfgang P. Ruf, Veselin Grozdanov, Axel Freischmidt, Marisa S. Feiler, Lisa Zondler, Albert C. Ludolph, Pamela J. McLean, Jochen H. Weishaupt, Karin M. Danzer

**Affiliations:** Department of Neurology, Ulm University, Albert-Einstein-Allee 11, 89081 Ulm, Germany; Mayo Clinic, Jacksonville, Florida USA

**Keywords:** Alpha synuclein, SNCA, SOD1, ALS, Parkinson’s disease, Cross-seeding, Oligomers

## Abstract

**Background:**

Parkinson’s disease (PD) and amyotrophic lateral sclerosis (ALS) are both neurodegenerative diseases leading to impaired execution of movement. α-Synuclein plays a central role in the pathogenesis of PD whereas Cu, Zn superoxide dismutase (SOD1) is a key player in a subset of familial ALS cases. Under pathological conditions both α-synuclein and SOD1 form oligomers and fibrils. In this study we investigated the possible molecular interaction of α-synuclein and SOD1 and its functional and pathological relevance.

**Results:**

Using a protein-fragment complementation approach and co-IP, we found that α-synuclein and SOD1 physically interact in living cells, human erythrocytes and mouse brain tissue. Additionally, our data show that disease related mutations in α-synuclein (A30P, A53T) and SOD1 (G85R, G93A) modify the binding of α-synuclein to SOD1. Notably, α-synuclein accelerates SOD1 oligomerization independent of SOD1 activity.

**Conclusion:**

This study provides evidence for a novel interaction of α-synuclein and SOD1 that might be relevant for neurodegenerative diseases.

**Electronic supplementary material:**

The online version of this article (doi:10.1186/s13024-015-0062-3) contains supplementary material, which is available to authorized users.

## Background

Parkinson’s disease (PD) is characterized by a progressive loss of dopaminergic neurons in the *substantia nigra pars compacta* and intracellular protein inclusions termed Lewy bodies whose main component is α-synuclein [1, 2]. Several point mutations have been reported in α-synuclein: A53T, A30P, E46K and H50Q, all of which result in familial forms of PD [3–7]. Therefore α-synuclein plays a key role in the pathogenesis of PD. The function of α-synuclein is complex involving the regulation of neurotransmitter release, exocytosis and trafficking of synaptic vesicles and co-chaperone activity [8–12]. α-Synuclein physiologically exists as an unfolded or membrane-bound monomer but is capable of forming oligomers, fibrils and finally inclusion bodies under pathological conditions [2, 13]. Notably, increasing evidence points to α-synuclein oligomers rather than fibrils as the toxic species leading to neurodegeneration [14–17].

ALS is another neurodegenerative disease that is characterized by a progressive loss of the upper and lower motor neurons resulting in spasticity and paresis. SOD1 is directly associated with a familial form of ALS with more than 100 different mutations in the SOD1 linked to ALS [18, 19]. SOD1 physiologically dimerizes and forms a non-covalently bound homodimer catalyzing the oxidation of O_2_˙^−^ to H_2_O_2_ or O_2_ [20]. Like α-synuclein, SOD1 pathologically aggregates forming soluble oligomers, insoluble fibrils and inclusion bodies [21–23].

The co-occurrence of ALS and PD has been reported on Guam and in the Kii Peninsula of Japan and several cases have also been described apart from the Western Pacific [24–30]. Additionally, extrapyramidal symptoms due to nigrostriatal dysfunction have been reported in ALS patients [31, 32] indicating that PD related pathological features may play a role in ALS. Indeed, several studies suggest an involvement of α-synuclein in ALS, for instance prominent phosphorylated α-synuclein inclusions were found in ALS-PD complex in Kii Peninsula [33]. In addition to the Kii Peninsula, other ALS cases with α-synuclein inclusions have been reported [25, 34, 35]. Furthermore, increased α-synuclein expression was identified in glial cells and in spheroids of the spinal cord of ALS patients and in the anterior horn cells of the spinal cord, in the hippocampus and cerebellum of mice expressing mutated SOD1 (G93A) [36, 37].

Although the literature, mentioned above, suggest an involvement of α-synuclein in ALS and a few studies have found that α-synuclein and SOD1 co-localize in the same protein aggregates [34, 38], hardly anything is known about a possible molecular α-synuclein-SOD1 interaction.

Therefore we asked whether SOD1 and α-synuclein directly interact and influence their respective oligomerization processes. To address these questions, we used human material, a mouse model for PD, and a PD cell culture model to determine the molecular interaction of α-synuclein and SOD1 and the impact of disease associated mutants of either α-synuclein or SOD1 on the interaction. Furthermore, using an α-synuclein or SOD1 protein complementation assay, we explored whether α-synuclein or SOD1 directly influence their oligomerization characteristics and exert cross-seeding activities.

## Results

### Detection of α-synuclein and SOD1 interaction in living cells, medium and ex vivo using a protein complementation approach

**Fig. 1:**
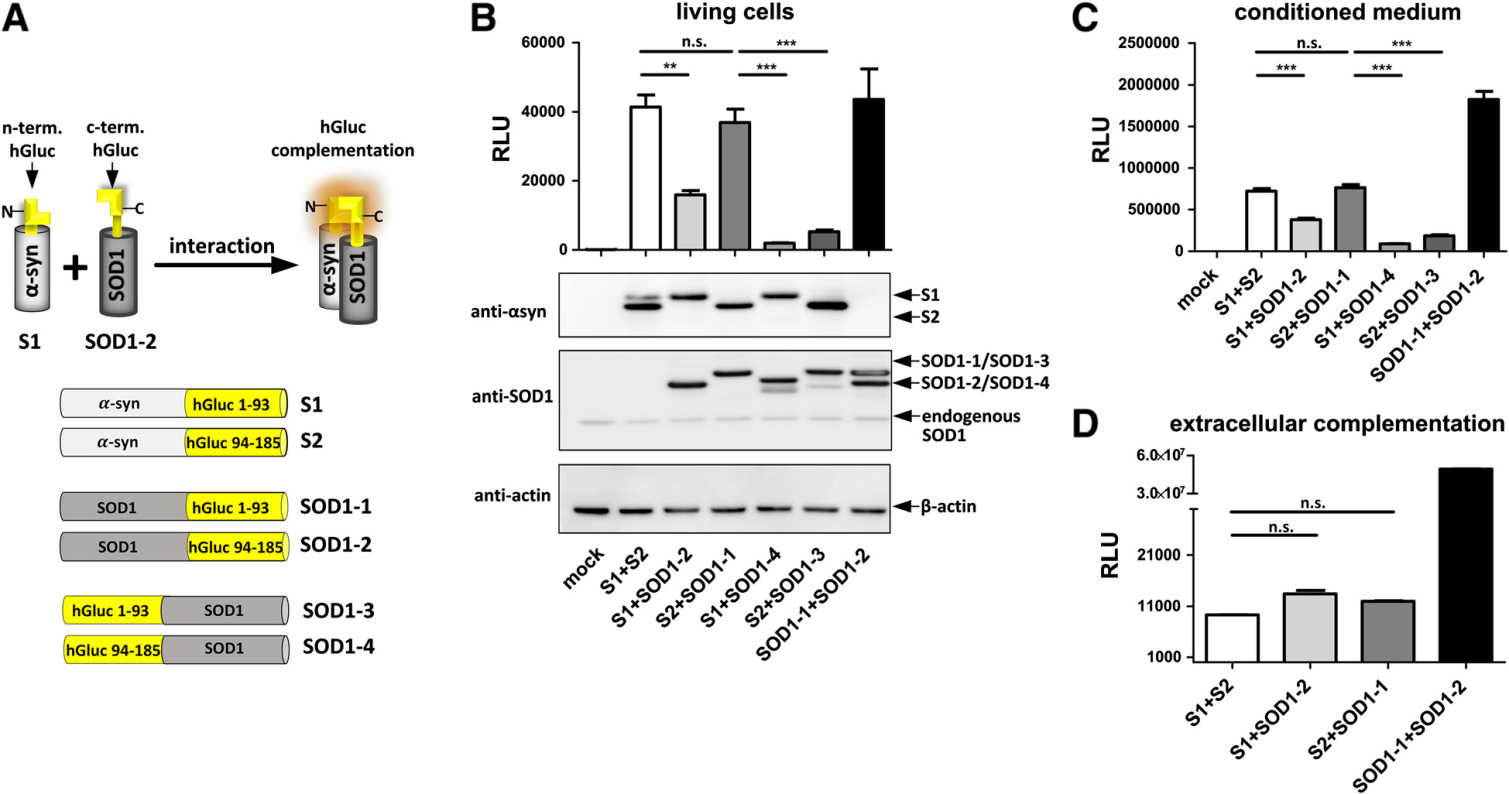
Detection of α-synuclein and SOD1 interaction using *Gaussia* luciferase (hGluc) protein-fragment complementation assay. **a** hGluc protein-fragment complementation principle: hGluc halves fused to the c-terminus of α-synuclein and SOD1 (S1, S2, SOD1-1, SOD1-2) or to the n-terminus of SOD1 (SOD1-3, SOD1-4) can restore luciferase activity due to protein-protein interactions. **b** Luciferase activity and western blot analysis (12 μg protein/lane) of H4 cells and **c** luciferase activity of conditioned medium 48 h post transfection (one way ANOVA, Tukey’s multiple comparison test, *n* = 6). **d** Extracellular complementation assay with H4 cell lysates containing 10,98 nM α-synuclein or SOD1. 12 h after combining the lysates, luciferase activity was measured (one way ANOVA, Tukey’s multiple comparison test, *n* = 3). Figures show representatively data from one experiment. Experiments have been repeated at least 2 times and showed similar results. (n.s. = not significant, * *p* < 0,05, ** *p* < 0,005, *** *p* < 0,0005) RLU = relative light unit

To investigate if α-synuclein and SOD1 interact at the molecular level, we used a *Gaussia* luciferase protein-fragment complementation assay. This assay monitors protein-protein interactions in living cells in real time and has already been described in detail for α-synuclein interactions [39, 40]. In this study, α-synuclein and SOD1 plasmid constructs tagged either with the n-terminal part (S1, SOD1-1 respectively) or with the c-terminal part (S2, SOD1-2 respectively) of *Gaussia* luciferase (hGluc) were co-expressed in human H4 neuroglioma cells. The presence of α-synuclein and SOD1 interactions results in hGluc enzyme activity (Fig. 1a). Interestingly, strong luciferase activity was measured in cells and conditioned medium of cells co-transfected with S1/SOD1-2 or S2/SOD1-1 (Fig. 1b and c; Additional file 2: Figure S2). By contrast, luciferase signal was absent in H4 cells co-transfected with hGluc halves alone (L1 + L2) demonstrating that n- or c-terminal fragments of hGluc cannot reconstitute nonspecifically (Additional file 2: Figure S2). These results suggest a specific intra- and extracellular interaction between α-synuclein and SOD1.

To determine if a specific conformation is required for α-synuclein and SOD1 to interact, SOD1 tagged with hGluc on its n-terminus (SOD1-3, SOD1-4) was co-expressed with S1 or S2. Notably, less luciferase activity was measured when H4 cells were co-transfected with tagged α-synuclein together with n-terminal hGluc tagged SOD1 (SOD1-3, SOD1-4) instead of c-terminus tagged SOD1 (SOD1-1, SOD1-2) (Fig. 1b and c) suggesting that a parallel conformation of both proteins favors co-aggregation. To ensure equal protein levels, western blots with α-synuclein and SOD1 specific antibodies confirmed that higher luciferase activity of S1/SOD1-2 or S2/SOD1-1 than S1/SOD1-4 and S2/SOD1-3 was not due to different expression levels (Fig. 1b).

To further validate our results, we performed an ex vivo experiment whereby lysates containing S1 or S2 alone were mixed with lysates containing SOD1-2 or SOD1-1 alone (see Additional file 1: Figure S1). Interestingly, luciferase activity reconstituted ex vivo (Fig. 1d), further suggesting that α-synuclein and SOD1 might also interact in the extracellular space. Altogether, these results indicate that α-synuclein and SOD1 bind to each other in a special conformation both in co-transfected H4 cells, conditioned medium and ex vivo.

### α-synuclein binds to SOD1 in cell lines, mouse brain and human erythrocytes

**Fig. 2:**
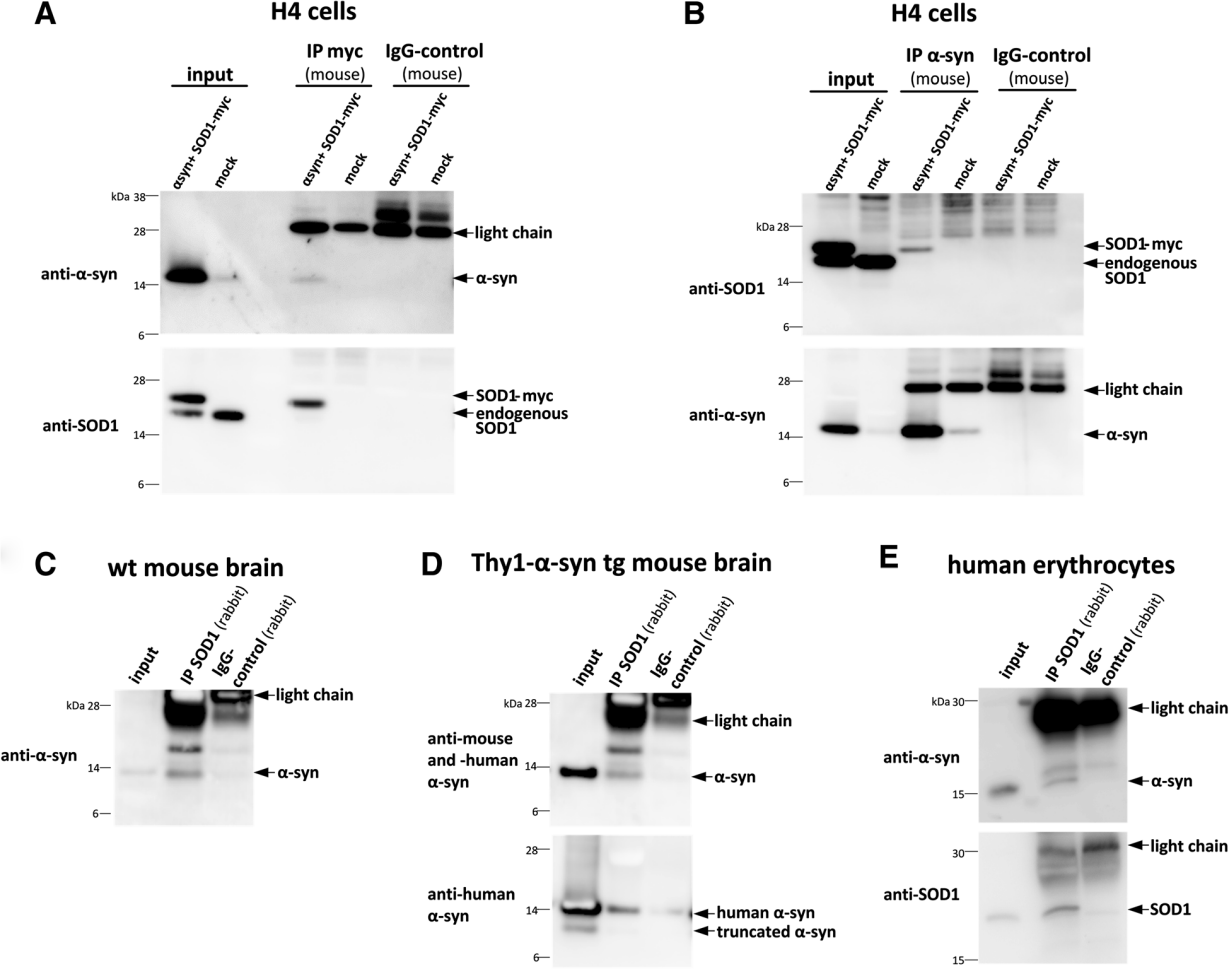
α-synuclein and SOD1 binding in cellular, mouse and human samples. **a** α-synuclein was detected when SOD1-myc was immunoprecipitated from co-transfected H4 cells using anti-myc antibody. Input: 5 μg protein. **b** α-Synuclein immunoprecipitation of co-transfected H4 cells co-immunoprecipitated SOD1-myc. Input: 5 μg protein. **c** α-synuclein was detected when SOD1 was immunoprecipitated from whole brain lysates of BDF1 wt mouse using anti-SOD1 antibody. Input: 10 μg protein. **d** SOD1 immunoprecipitation of whole brain lysates of Thy1-α-synuclein tg mouse co-immunoprecipitated mouse and human α-synuclein. Input: 10 μg protein. **e** SOD1 immunoprecipitation of human erythrocytes using rabbit-anti-SOD1 antibody of human erythrocytes detects α-synuclein using mouse-anti-α-synuclein antibody. Input: 20 μg protein. IgG control: lysates were incubated with unspecific IgG-antibodies derived from the same species as the primary antibody. light chain = light chain of IgGs

To confirm the interaction of α-synuclein and SOD1 with a different and luciferase independent method, we performed co-immunoprecipitations (co-IPs) with α-synuclein and SOD1-myc co-transfected cells. Importantly, α-synuclein was detected when SOD1-myc was immunoprecipitated from co-transfected H4 cells (Fig. 2a) whereas the IgG control did not pull down α-synuclein. The reverse co-IP of α-synuclein (mouse anti-α-synuclein antibody) co-immunoprecipitated SOD1-myc (Fig. 2b) and endogenous SOD1 (Additional file 3: Figure S3A) whereby mouse IgG did not precipitate SOD1-myc or endogenous SOD1. Taken together, co-IP studies further confirmed that α-synuclein and SOD1 physically interact in co-transfected cells, consistent with the results of the hGluc protein-fragment complementation assay.

To demonstrate α-synuclein and SOD1 interaction in vivo and to underline its physiological relevance, we performed co-IPs from whole brain lysates of Thy1-α-synuclein tg mice and wild-type (wt) mice. As demonstrated in Fig. 2c and d, we could confirm a physical interaction of α-synuclein and SOD1 in brains of wt mice and in the α-synuclein tg mice. We also found a cross-species interaction between human α-synuclein and mouse SOD1 (Fig. 2d).

To further validate our findings on α-synuclein and SOD1 interaction, we performed co-IPs with human erythrocytes since they contain high amounts of both α-synuclein and SOD1 and are easily accessible. Most importantly, α-synuclein could be detected when SOD1 was immunoprecipitated with an SOD1 specific antibody (Fig. 2e). Thus the interaction between α-synuclein and SOD1 could be further verified in human erythrocytes.

**Fig. 3:**
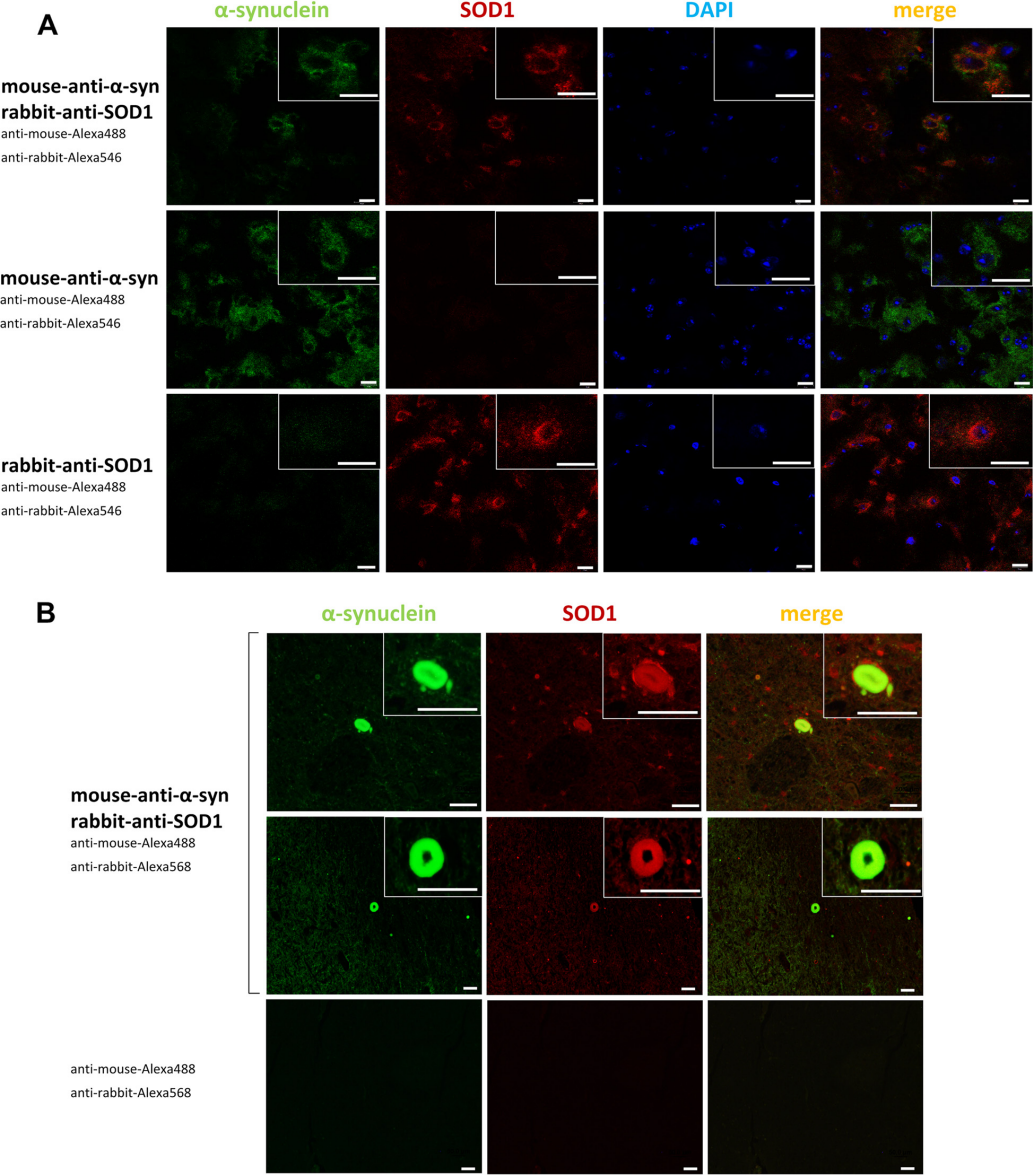
Co-localization of α-synuclein and SOD1 in wt mouse and human brain. **a** Representative confocal microscopy images of C57Bl/6 wt mouse brain sections co-immunostained for α-synuclein (Alexa 488, green) and SOD1 (Alexa 546, red). Nuclei were stained with DAPI. As control, single staining of α-synuclein and SOD1 was performed with respective primary antibody alone and with both fluorophores (Alexa 488, Alexa 546) conjugated secondary antibodies. **b** Representative microscopy images of human midbrain tissue co-immunostained for α-synuclein (Alexa 488, green) and SOD1 (Alexa 568, red). As control, sections were stained with both conjugated secondary antibodies (Alexa 488, Alexa 568) without primary antibodies

We also used immunhistochemistry (IHC) to identify co-localization of SOD1 and α-synuclein in wt mouse brain tissue and in human brain tissue of a patient with dementia with Lewy bodies (DLB). Confocal microscopy (Fig. 3a) and fluorescent microscopy (Additional file 4: Figure S4) show a clear colocalization of α-synuclein and SOD1 in mouse brain tissue sections. Single staining controls, with either α-synuclein or SOD1 antibody alone but with both secondary antibodies, demonstrate that co-localization of α-synuclein and SOD1 did not arise due to a nonspecific crossover artifact. Additionally, immunostaining of α-synuclein and SOD1 in midbrain tissue sections of a DLB case reveals co-localization of α-synuclein and SOD1 in protein aggregates (Fig. 3b).

In sum, IHC shows a local correlation of α-synuclein and SOD1 in mouse and human brain tissue which could be confirmed by co-IP studies detecting molecular interaction of α-synuclein and SOD1.

### PD related mutations in α-synuclein alter the interaction of α-synuclein and SOD1

**Fig. 4:**
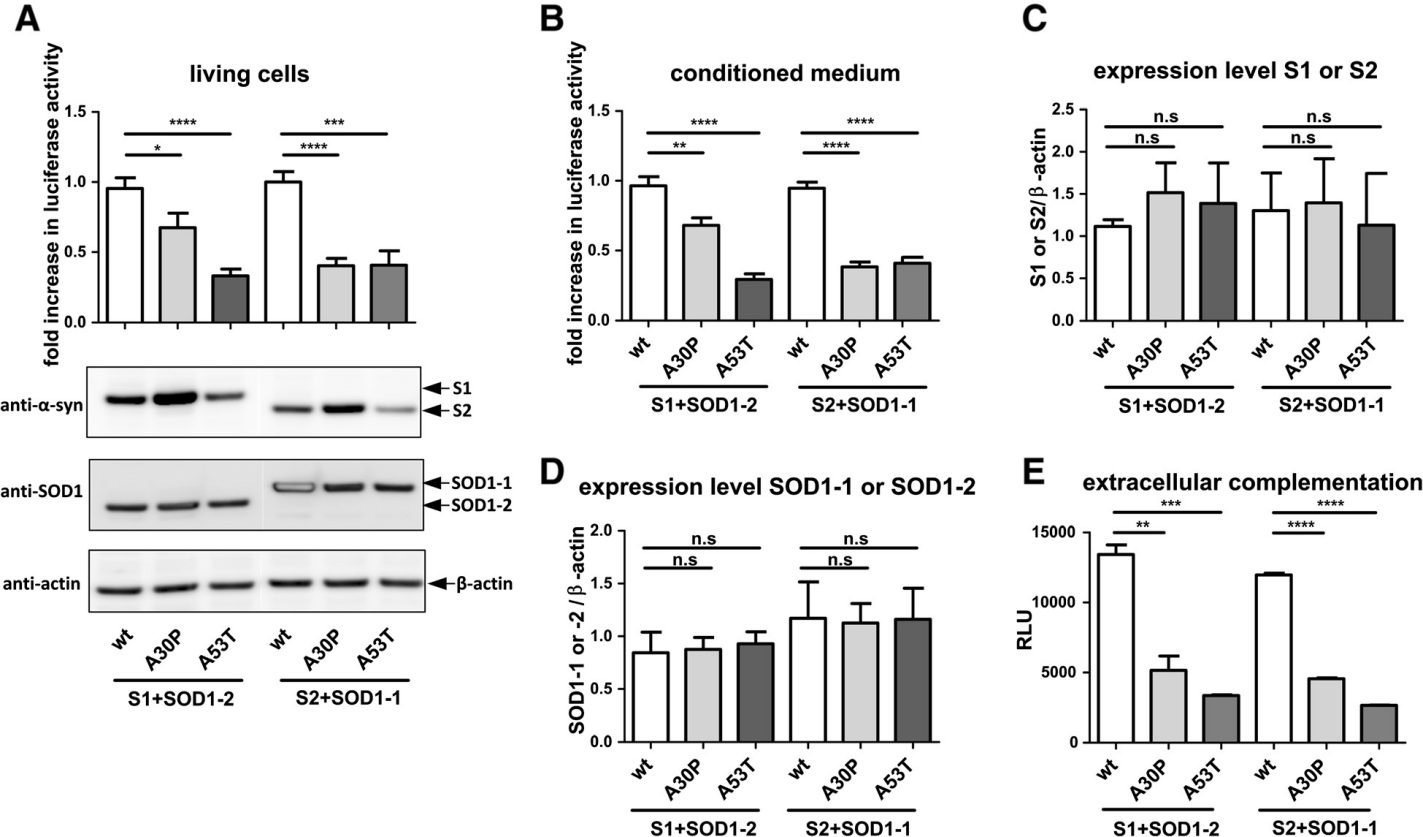
Pathological α-synuclein mutations (A30P, A53T) alter the α-synuclein-SOD1 interference. **a** Luciferase activity measurement and western blot analysis (12 μg protein/lane) of H4 cells co-transfected with hGluc tagged wt, A30P or A53T α-synuclein and hGluc tagged SOD1. Data from 3 independent experiments were pooled after normalization to the respective mean of luciferase activity of wt α-synuclein (two tailed, unpaired student’s t-test, *n* = 9). **b** Luciferase activity of conditioned medium from cells in a. Shown are the pooled data from 3 independent experiments after normalization to mean of luciferase activity of co-transfected cells with wt α-synuclein (two tailed, unpaired student’s t-test, *n* = 6-9). **c** and **d** Densitometric analysis of western blots of co-transfected H4 cells. SOD1-1, SOD1-2, S1 and S2 were normalized to β-actin (two tailed, unpaired student’s t-test, *n* = 3). **e** Representative extracellular complementation assay (ECA) with H4 cell lysates containing 10,98 nM of wt, A30P or A53T α-synuclein or SOD1 is shown. 12 h after combining the lysates, luciferase activity was measured (two tailed, unpaired student’s t-test, *n* = 3). ECA was repeated 2 times and showed similar results. (n.s. = not significant, * *p* < 0,05, ** *p* < 0,005, *** *p* < 0,0005, **** *p* < 0,0001) RLU = relative light unit

To determine whether PD linked mutations in α-synuclein modify the α-synuclein-SOD1 interaction, we performed a hGluc protein-fragment complementation assay in cells and conditioned media expressing SOD1 and α-synuclein containing the A30P or A53T mutation, respectively. Strikingly, we found a reduction in luciferase activity with A30P or A53T α-synuclein compared to wt α-synuclein (Fig. 4a and b). Western blot analysis with α-synuclein and SOD1 specific antibodies showed that reduced luciferase activity of A30P-S1 and A53T-S1 was not a result of different expression levels (Fig. 4c and d). To confirm reduced interaction of α-synuclein mutants independent of cellular environment, we conducted an extracellular complementation assay with equal amounts of wt, A30P and A53T α-synuclein. In this assay the molarity of wt, A30P and A53T α-synuclein was normalized using an α-synuclein specific ELISA before protein-protein interaction ex vivo was measured (see Additional file 1: Figure S1). In accord with the experiment performed in living cells, significantly less luciferase activity was detected when SOD1 was incubated with A30P or A53T α-synuclein compared to wt α-synuclein (Fig. 4e) suggesting that A30P and A53T α-synuclein mutations have a lower binding affinity to SOD1 than wt α-synuclein.

### Familial ALS mutations in SOD1 affect the binding of SOD1 to α-synuclein

**Fig. 5:**
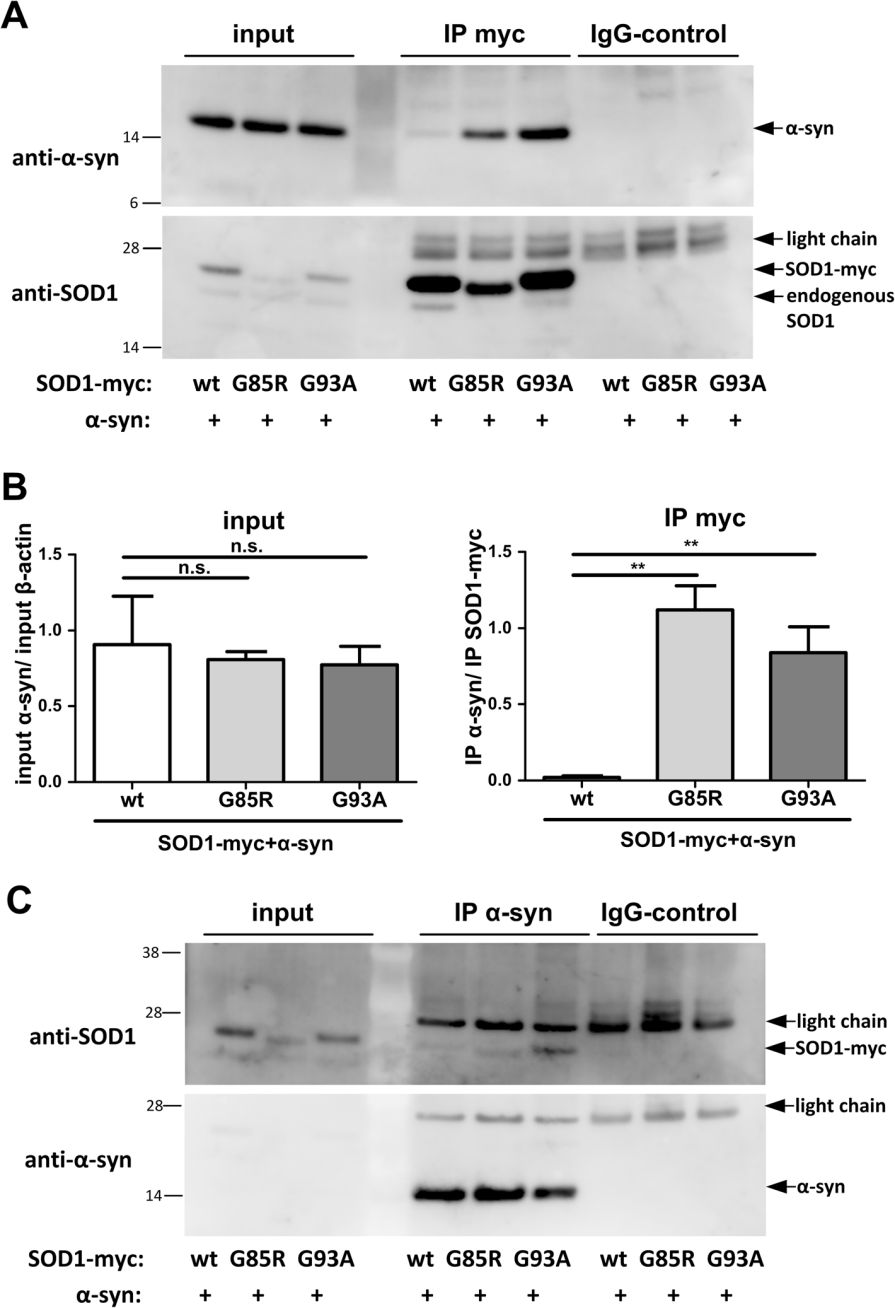
Pathological G85R and G93A mutations in SOD1 alter binding affinity to α-synuclein. **a** Immunoprecipitation of G85R and G93A SOD1-myc of co-transfected H4 cells using anti-myc antibody increasingly precipitated α-synuclein. Input: 5 μg protein. Experiment was repeated 4 times and showed similar results. **b** Quantification of SOD1-myc immunoprecipitations. Co-immunoprecipitated α-synuclein was normalized to immunoprecipitated wt, G85R and G93ASOD1-myc respectively. α-synuclein of input was normalized to β-actin of input (two tailed, unpaired student’s t-test, *n* = 3, n.s. = not significant, * *p* < 0,05). **c** α-synuclein immunoprecipitation of co-transfected H4 cells co-immunoprecipitated particularly G85R and G93A SOD1-myc. Input: 5 μg protein. Experiment was repeated 2 times with similar results. light chain = light chain of IgGs

Conversely we investigated if familial ALS related mutations in SOD1 influence the binding of SOD1 to α-synuclein. Because G85R and G93A SOD1 are unstable and thereby expressed at lower levels than wt SOD1, we used co-IP, instead of the luciferase assay, to examine the effect of G85R and G93A mutations in SOD1 on α-synuclein binding. Interestingly, more α-synuclein co-immunoprecipitated with G85R or G93A SOD1-myc compared to wt SOD1-myc (Fig. 5a). Quantification of the co-IPs revealed a significant increase in α-synuclein interaction with G93A and G85R SOD1-myc compared to wt SOD1-myc (Fig. 5b). Reversely, similar results were obtained when α-synuclein specific antibodies were used to pull down α-synuclein. More mutated SOD1-myc was co-immunoprecipitated compared to wt SOD1-myc in the α-synuclein immunoprecipitation experiments whereas the amount of immunoprecipitated α-synuclein was similar (Fig. 5c). Taken together, our results suggest that G85R and G93A SOD1 binds with higher affinity to α-synuclein compared to wt SOD1.

### α-synuclein does not influence the enzymatic activity of SOD1

**Fig. 6:**
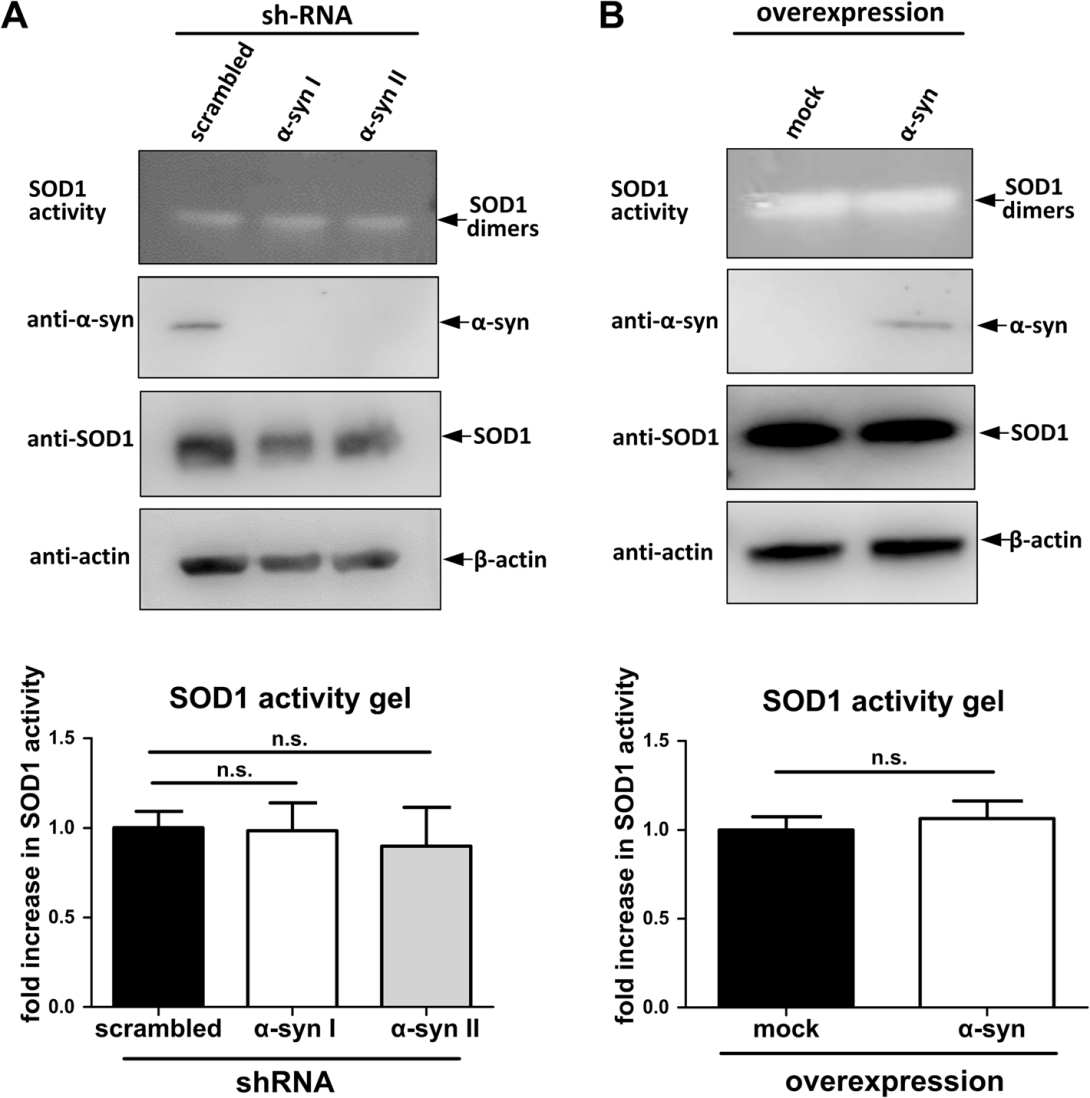
α-synuclein does not influence enzymatic activity of SOD1. **a** SOD1 activity gel (20 μg protein/lane) and western blot analysis (40 μg protein/lane) of stable scrambled-shRNA H4 cell line and two stable α-synuclein knockdown H4 cell lines (shRNA α-syn I and α-syn II) (two tailed, unpaired student’s t-test, *n* = 5). **b** SOD1 activity gel (20 μg protein/lane) and western blot (18 μg protein/lane) analysis of mock and α-synuclein transient transfected H4 cells (two tailed, unpaired student’s t-test, *n* = 4). (n.s. = not significant)

Because α-synuclein has been suggested to have chaperone activity [12, 41], we asked if α-synuclein may act as a chaperone for SOD1. To examine the influence of α-synuclein on the enzymatic activity of SOD1, we performed SOD1 activity gels [42] with α-synuclein-overexpressing and -knockdown H4 cells. As shown in Fig. 6b, the transient overexpression of α-synuclein in H4 cells does not influence the SOD1 activity. In accord with this result, the enzymatic activity was not altered when α-synuclein expression was silenced using lentiviral delivery of α-synuclein shRNAs (α-syn shRNA I and α-syn shRNA II) leading to stable α-synuclein knockdown H4 cell lines (Fig. 6a). Together, α-synuclein seems to have no influence on the enzymatic activity of SOD1.

### α-synuclein accelerates SOD1 dimerization

**Fig. 7:**
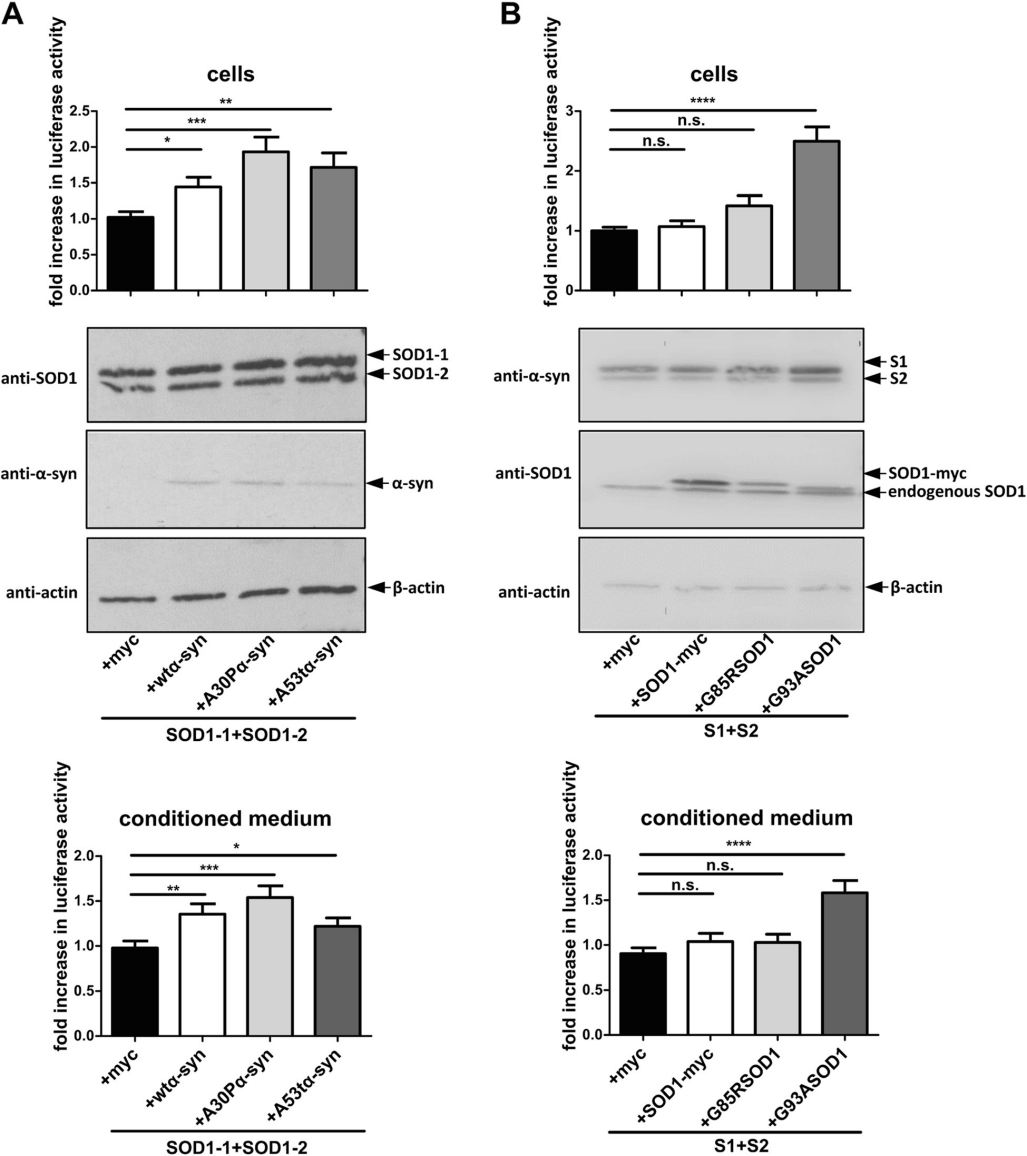
Mutual influence of α-synuclein and SOD1 on their dimerization/oligomerization. **a** Luciferase activity and western blot analysis (20 μg protein/lane) of H4 cell lysates and conditioned medium 48 h after transfection. SOD1-1 and SOD1-2 were expressed along with myc, wt, A30P or A53T α-synuclein. Data from 3 independent experiments were pooled after normalization to mean luciferase activity of SOD1-1 + SOD1-2 + myc (two tailed, unpaired student’s t-test, *n* = 33-36). **b** α-synuclein oligomerization in the presence of myc, wt, G85R or G93A SOD1-myc. 48 h post transfection, luciferase assay and western blot analysis with cell lysates (5 μg protein/lane) and conditioned medium. Data from 7 independent experiments were pooled after normalization to respective mean of the luciferase activity of S1 + S2 + myc (two tailed, unpaired student’s t-test, *n* = 83-84). (n.s. = not significant, * *p* < 0,05, ** *p* < 0,005, *** *p* < 0,0005, **** *p* < 0,0001)

**Fig. 8:**
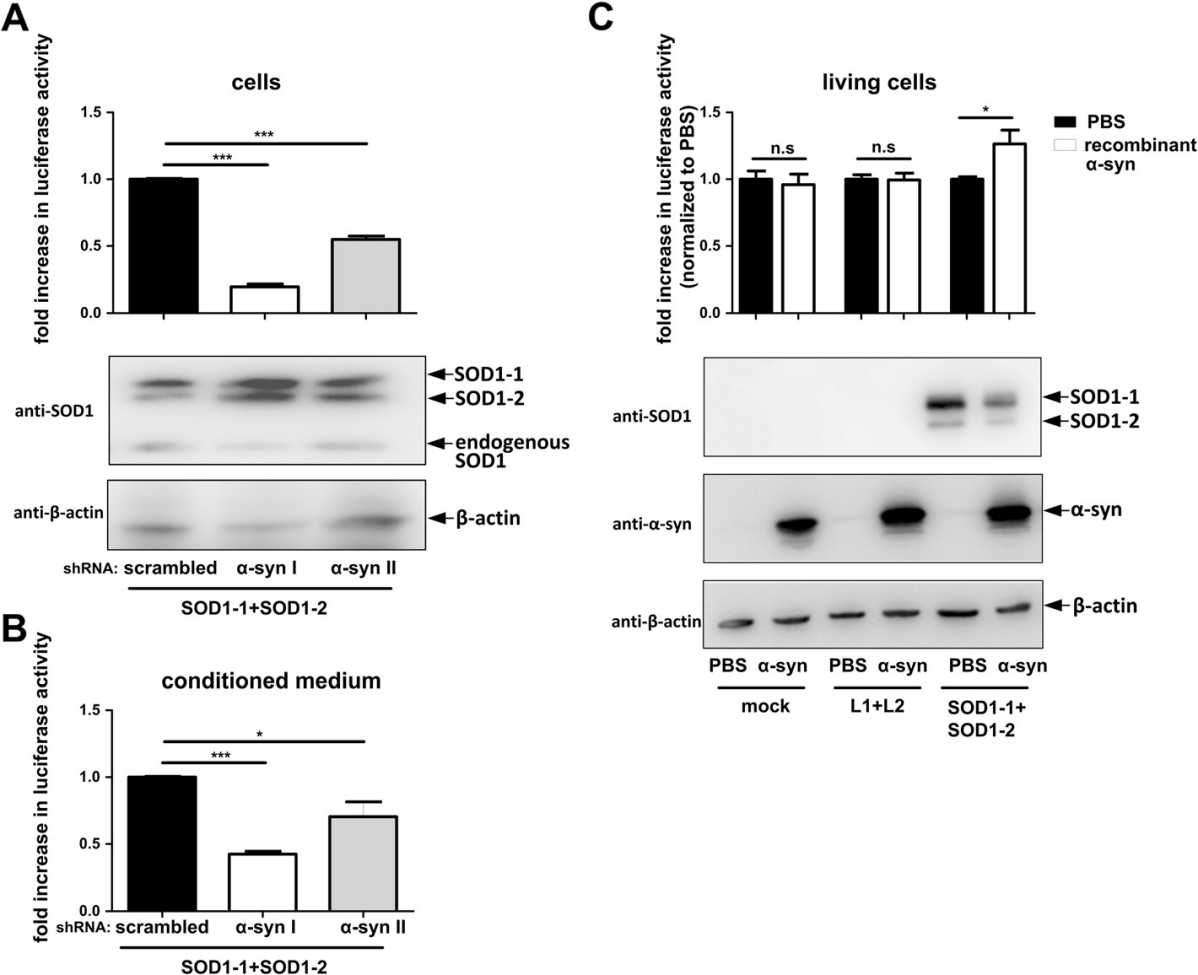
α-synuclein increases SOD1 oligomerization. **a** Luciferase activity and representative western blot of H4 cells (18 μg protein/lane) and **b** luciferase activity of conditioned medium of scrambled-shRNA stabile H4 cell line and two α-synuclein-shRNA stabile H4 cell lines (α-syn I, α-syn II) co-transfected with SOD1-1 and SOD1-2. Figures show pooled data from 4 independent experiments after normalization to respective mean luciferase activity of scrambled-shRNA cells (two tailed, unpaired student’s t-test, *n* = 8-11). **c** Luciferase activity measurement and representative western blot (5 µg protein/lane) of transfected H4 cells incubated with 7 μM recombinant α-synuclein or solvent control PBS. Data from 3 independent experiments were pooled after normalization to the respective PBS control (two tailed, unpaired student’s t-test, *n* = 10-12). (n.s. = not significant, * *p* < 0,05, *** *p* < 0,0005)

We, and others, have previously shown a prion-like seeding process for α-synuclein [17, 43, 44]. Cross-seeding effects of α-synuclein with other relevant neurodegenerative disease proteins like tau and amyloid-β have already been described [45, 46]. To determine whether α-synuclein impacts SOD1 oligomerization, α-synuclein was co-transfected with SOD1-1 and SOD1-2. Here, an increase in SOD1 dimerization was detected when wt, A30P or A53T α-synuclein was co-transfected together with SOD1 (Fig. 7a) whereas the expression level of SOD1-1 and SOD1-2 was not altered (Additional file 5: Figure S5a) suggesting that α-synuclein increases SOD1 dimerization and/or oligomerization. Accordingly, we asked whether α-synuclein knockdown would reduce oligomerization of SOD1. Stably α-synuclein down-regulated H4 cells transfected with SOD1 -1/-2 complementation pair had an at least 2,1 fold decrease in SOD1 dimerization (Fig. 8a) further supporting the finding that α-synuclein enhances SOD1 dimerization and/or oligomerization. As an additional control, recombinant α-synuclein was added to SOD1-1 and SOD1-2 co-transfected H4 cells and incubated for 24 h. As demonstrated in Fig. 8c, also recombinant α-synuclein increases SOD1 dimerization compared to solvent PBS treated cells whereas recombinant α-synuclein did not enhance luciferase activity in mock and L1/L2 transfected cells. Western blot analysis with α-synuclein and SOD1 specific antibodies showed that increased  luciferase activity of recombinant α-synuclein was not a result of different expression levels (Additional file 5: Figure S5d).

*Vice versa* we examined if SOD1 promotes α-synuclein oligomerization thereby measuring luciferase activity of H4 cells expressing S1 and S2 with wt or mutated SOD1. We found that wt and G85R SOD1 did not influence the oligomerization of α-synuclein (Fig. 7b). By contrast, G93A SOD1 dramatically increases the α-synuclein oligomerization.

Collectively, these data indicate that α-synuclein and SOD1 mutually exacerbate each other’s aggregation properties with disease-related mutations of both proteins having stronger effects.

## Discussion

This study provides evidence for a novel interaction between α-synuclein and SOD1. Using a *Gaussia* luciferase protein fragment complementation assay and immunoprecipitation approaches, we found α-synuclein-SOD1 interaction in α-synuclein and SOD1 transfected cells and in conditioned medium, but also in mouse brain tissue and human erythrocytes. Our results from the *Gaussia* luciferase protein-fragment complementation assay show that there is a high degree of α-synuclein-SOD1 interaction which is comparable to α-synuclein oligomerization (S1 + S2) indicating that α-synuclein-SOD1 binding might have a comparable binding affinity and kinetic to α-synuclein oligomerization. Since endogenous SOD1 might compete with hGluc tagged SOD1 for the binding to hGluc tagged α-synuclein, the SOD1 and α-synuclein interaction might be even more pronounced in vivo. Furthermore, our microscopy data support the finding of α-synuclein-SOD1 interaction by detecting a co-localization of SOD1 and α-synuclein in wt mouse and human brain tissue. The interaction of α-synuclein and SOD1 could also be proven with co-IPs of co-transfected cells, mouse brains of wt and Thy1-α-synuclein tg mice and human erythrocytes. These findings suggest that the α-synuclein-SOD1 interaction might take place in all organs co-expressing α-synuclein and SOD1. Since SOD1 is ubiquitously expressed and α-synuclein occurs in all organs tested so far except the liver [47, 48], the α-synuclein-SOD1 binding might take place in other organs, too.

As a first step towards understanding the molecular consequence of the α-synuclein and SOD1 interaction and its relevance to human diseases, we investigated if PD related mutations in α-synuclein and ALS related mutations in SOD1 modify the α-synuclein-SOD1 interaction. The results of the luciferase assay show that familiar A30P and A53T α-synuclein mutations have reduced interaction capabilities with SOD1 compared to wt α-synuclein in living cells and conditioned medium. The extracellular complementation assay where the same molarity of mutated and wt hGluc tagged α-synuclein is used confirms the reduced interaction of familiar A30P and A53T α-synuclein mutations to SOD1. In contrast to familial α-synuclein mutation, our co-IP studies show that G85R and G93A SOD1 known to cause familial ALS have a higher tendency to bind α-synuclein compared to wt SOD1. As expression levels of G85R, G93A and wt SOD1 are not equal, we considered co-IP as an appropriate method to examine the influence of disease related mutations in SOD1. We observed that G85R and G93A SOD1 increasingly bind to α-synuclein compared to wt SOD1. Taken together, our results show that pathological mutations in α-synuclein and SOD1 alter the interaction of α-synuclein and SOD1 suggesting a relevance of the interaction of α-synuclein and SOD1 in human diseases.

In addition to our findings on a molecular interaction of α-synuclein and SOD1, we also investigated whether α-synuclein and SOD1 influence their oligomerization capabilities. Recent studies highlight cross-seeding activities where one protein accelerates the aggregation of other proteins involved in neurodegeneration. Interestingly, we found that α-synuclein accelerates SOD1 dimerization. Consequently, reduced SOD1 dimerization was detected in α-synuclein knockdown cell lines. SOD1 homodimers are enzymatic active whereas aggregates of SOD1 seem to have a reduced enzyme activity [49]. Since we did not find an influence of α-synuclein on the enzymatic activity of SOD1, we conclude that α-synuclein increases the SOD1 oligomerization and higher molecular weight species rather than solely SOD1 dimerization.

Seeding effects of α-synuclein to other aggregation prone proteins by a prion-like principle is in accordance with other studies [43, 44]. Notably, a cross-seeding activity for α-synuclein with other aggregating proteins has already been described: α-synuclein accelerates the aggregation of tau and amyloid-β, proteins implicated in Alzheimer’s disease (AD), and huntingtin, a protein involved in Huntington’s disease [45, 46, 50–53]. The seeding activity of α-synuclein to tau has even been shown in vivo [51]. Future in vivo studies will be needed to investigate if the α-synuclein is also able to promote SOD1 oligomerization in vivo.

Moreover, our data indicate that SOD1 oligomerization is even more enhanced in presence of A30P or A53T α-synuclein compared to wt α-synuclein. A plausible explanation would be that more unbound SOD1 is available because A30P and A53T α-synuclein bind less to SOD1 than wt α-synuclein.

Notably, SOD1 with G93A mutation, but not G85R and wt SOD1, was able to promote the α-synuclein oligomerization. Our findings could be a possible explanation why increased α-synuclein was found in spinal cord, hippocampus and cerebellum of mice expressing G93A SOD1 compared to mice expressing wt SOD1 [37]. Interestingly, spinal cord homogenate of G93A tg mice have been shown to accelerate SOD1 aggregation formation in vitro and in vivo and to mediate the transmission of motor symptoms [54, 55]. Thus, our study supports previous studies demonstrating seeding properties of G93A SOD1.

Although we show that α-synuclein co-localizes with SOD1 in protein aggregates and promotes the SOD1 aggregation, there are only few studies showing α-synuclein and SOD1 in the same protein inclusions [34, 38]. It might be possible that the interaction of SOD1 and α-synuclein is disrupted in diseases or α-synuclein seeds SOD1 without being internalized into protein inclusions. Thus, future studies in a larger number of patient post-mortem material are needed to determine if α-synuclein and SOD1 co-occur in the same protein aggregates.

## Conclusion

Our study provides the first evidence for a direct interaction between α-synuclein and SOD1 suggesting common pathogenic pathways in ALS and PD. We detected an interaction of α-synuclein and SOD1 in different cell types and tissue: human neuroglioma H4, human erythrocytes and mouse and human brain tissue. Interestingly, PD related mutations in α-synuclein and familiar ALS mutations in SOD1 change the binding of both proteins suggesting that this interaction might be altered in human diseases. The functional mechanism of the interplay of α-synuclein and SOD1 seems to be independent of a role of α-synuclein affecting SOD1 activity. α-Synuclein might rather increase SOD1 oligomerization potentially leading to aberrant SOD1 aggregation. This work has implications on the understanding on both neurodegenerative diseases, ALS and PD, and might suggest a coherent consideration and study of the key players in neurodegenerative diseases.

## Methods

### Plasmid generation

Fusion constructs α-synuclein-hGluc1 (S1) and α-synuclein-hGluc2 (S2) are based on pcDNA3.1-Zipper-hGluc(1) and pcDNA3.1-Zipper-hGluc(2) and have been described previously [40, 56]. α-synuclein was replaced by SOD1 to generate SOD1-hGluc1 (SOD1-1) and SOD1-hGluc2 (SOD1-2). Fusion constructs SOD1-3 and SOD1-4 were created by cloning hGluc1 or hGluc2, respectively, with a linker to the n-terminus of SOD1 into pcDNA3.

### Cell culture and transfection

H4 (human neuroglioma) were cultivated at 37 °C in 5 % CO_2_ in OptiMEM® or DMEM (both from Life technologies, Carlsbad, USA) supplemented with 10 % FBS (Sigma, St. Louis, USA; or Life technologies, Carlsbad, USA). Cells were transfected 24 h after plating with Superfect® (Quiagen, Chatsworth, USA) or Fugene6® (Promega, Fitchburg, USA) transfection reagent according to the manufacturers’ instructions. Cells were further cultivated at 37 °C in 5 % CO_2_ for 24 h.

### Cell lysis

Cells were washed with PBS and scraped from dishes on ice with cold TritonX lysis buffer (150 mM NaCl, 50 mM TrisHCl pH 7,4, 0,1 % TritonX) containing protease inhibitor (Roche Diagnostics). Different lysis buffers were used for co-IP (see below). Lysate was centrifuged at 10000 g for 10 min at 4 °C and total protein concentrations of the supernatants were determined with the BCA assay (Pierce Biotechnology, Rockford, USA) according to the manufacturer’s instructions.

### *Gaussia* luciferase protein-fragment complementation assay

The *Gaussia* luciferase protein-fragment complementation assay was performed either with transfected living cells in a 96-well plate, lysates of transfected cells or with conditioned medium. After transferring 100 μl of cultured medium to a new 96-well plate, living cells in 96-well plate were washed with PBS and 100 μl of a serum- and phenolred- free medium was added per well. Cell lysates were prepared as described above, adjusted to the same protein concentration and 100 μl of the adjusted cell lysates were added to each well of a 96-well plate. The luciferase activity was measured by a luminometer (Multilabel plate reader Victor3, Perkin Elmer) that automatically applied coelenterazine (Nanolight, Pinetop, USA), a cell permeable substrate of the luciferase, to a final concentration of 20 μM.

### Treatment of transfected H4 cells with recombinant α-synuclein

24 h post transfection, H4 cells in 96-well plates or 6 cm dishes were washed with PBS and treated with serum- and phenolred- free medium containing 7 μM of recombinant α-synuclein (Roche, Rotkreutz, Swiss) or the same volume of the corresponding solvent (PBS). After 24 h, *Gaussia* luciferase protein-fragment complementation assay with living cells in 96-well plates or western blot with cells in 6 cm dishes was performed.

### Extracellular complementation assay

To detect protein-protein binding ex vivo, we utilized extracellular complementation assay with lysates from H4 cells overexpressing either α-synuclein- or SOD1-hGluc alone. The concentrations of α-synuclein and SOD1 were quantified by ELISA (Invitrogen, Carlsbad, CA, USA; Enzo life science, New York, USA, respectively) and adjusted to the same molarity. α-synuclein/SOD1 fused to either luciferase half were mixed and incubated in a spinning wheel at 4 °C for 12 h before measuring luciferase activity.

### Western blotting

Proteins were separated by standard SDS-PAGE using the NuPAGE® system (Invitrogen, Carlsbad, CA, USA), followed by a transfer to PVDF membranes (Millipore, Billerica, USA). To improve the detection of endogenous α-synuclein of H4 cells, the membranes were fixed with 0,4 % PFA prior blocking as described previously [57]. Following antibodies were used: rabbit-anti-β-actin, (1:2000, Sigma, St. Louis, USA or 1:1000, abcam, Cambridge, UK), mouse-anti-α-synuclein (4B12, 1:3000, Covance, Princeton, USA), mouse-anti-α-synuclein (LB-509, 1:1000, Covance, Princeton, USA), rabbit-anti-SOD1 (ADI-SOD-100, 1:2000, Enzo life science, New York, USA), sheep-anti-SOD1 (1:1000, Calbiochem, Farmingdale, USA), HRP coupled secondary antibodies (1:1000, Life Technologies, Carlsbad, USA, or SouthernBioTech, Birmingham, USA).

### SOD1 activity gels

H4 cells were lysed in dH_2_O by 3 freeze-thaw cycles and centrifuged at 15000 g for 10 min at 4 °C. 20 μg total protein per lane, combined with 5x loading buffer (62,5 mM TrisHCl pH 6,8, 30 % Glycerol, 0,05 % bromphenol blue), were separated in 12 % non-reducing Tris-Glycin gel. Gel was stained, as described previously [42], in 2,45 mM nitro blue tetrazolium (NBT) solution for 20 min and then in developer solution (28 mM TEMED, 28 μM riboflavin, 36 mM KH_2_PO_4_ pH 7,4) for 15 min in darkness. Gel was illuminated until sufficient contrast between light line (SOD1 activity) and background was achieved.

### Co-immunoprecipitation from H4 cells

The co-immunoprecipitation (co-IP) was performed using a adapted version of the ReCLIP method (reversible cross-link immunoprecipitation) [58, 59]. In brief, H4 cells were washed 2 times with PBS 24 h post transfection. Then, lysine residues of the interacting proteins were crosslinked by incubation with 1 mM of the cell permeable dithiobissuccinimidylpropionate (DSP) at RT for 30 min. After incubation with quenching solution (20 mM TrisHCl pH 7,4; 5 mM L-cysteine in PBS) for 10 min and washing step with PBS, cells were lysed with NP-40 lysis buffer (150 mM NaCl, 10 mM TrisHCl pH 7,4, 1 mM EDTA, 1 mM EGTA, 0,5 % (v/v) NP-40, 5 % (v/v), complete mini protease inhibitor). Lysates were centrifuged at 10000 g for 10 min at 4 °C and adjusted to the same protein concentration with the respective lysis buffer. To remove unspecific protein binding to the beads, 20 μl Protein A and G mag sepharose Xtra magnetic beads (GE Healthcare, Chalfont St Giles, UK) were incubated with lysates for 1 h at 4 °C with end-over rotation and then separated from the lysates. Pre-cleared lysates were incubated with primary antibodies (mouse-anti-c-myc, Roche, Mannheim, Germany; mouse-anti-α-synuclein (4B12), Covance, Princeton, USA; rabbit-anti-SOD1 (ADI-SOD-100), Enzo life science, New York, USA; rabbit-anti-SOD1, gift from Dennis Dickson, Mayo Clinic Jacksonville [60]) or, as control, with IgGs from the same species as the primary antibody’s species (ChromPure Mouse IgG and ChromPure rabbit IgG, both Jackson Immuno, West Grove, USA) with end-over rotation overnight, followed by incubation with Protein G and A mag sepharose Xtra magnetic beads for 2 h on the next day. After washing the beads 3 times with lysis buffer, bound proteins were eluted and de-crosslinked by incubating with 40 μl of NuPAGE® LDL sample buffer (Invitrogen, Carlsbad, USA) and dithiothreitol (DTT) at a final concentration of 80 mM at RT for 15 min and then boiled at 95 °C for 5 min before loading on a gel.

### Co-IP from brain and erythrocytes

For co-IP with whole mouse brain homogenate, BDF1 wt mouse and Thy1-α-synuclein-tg mouse [61] (both 7 months old, female) were anaesthetized with ketamine and a 10 % (w/v) brain homogenate was made in PBS by mechanical homogenization. Human erythrocytes were separated from mononuclear blood cells by density gradient centrifugation of EDTA-whole blood on Histopaque (Sigma Aldrich, St. Louis, USA) at 500 g for 30 min at RT. Erythrocytes and brain homogenates were incubated with 10 mM DSP for 30 min, followed by lysis with same volume of TritonX lysis buffer (100 mM NaCl, 50 mM TrisHCl pH 7,4, 5 mM EDTA, 0,3 % v/v TritonX, 5 % (v/v), complete mini protease inhibitor) for 30 min on ice. For co-IP without crosslinking, lysis buffer was added directly to homogenized mouse brain. Afterwards, brain homogenate lysates were centrifuged at 9000 g for 10 min at 4 °C and co-IP was performed as described above. In case of erythrocyte lysates, antibodies were first coupled to the magnetic beads by incubating antibodies and beads in lysis buffer with 2 % BSA overnight at 4 °C with end-over rotation. Then, the antibody coupled beads were incubated with pre-cleared lysates at 4 °C for 2 h, followed by the standard co-IP protocol.

### Generation of stabile α-synuclein knockdown cell lines

Endogenous α-synuclein was knocked down by shRNA to establish two stable α-synuclein knockdown cell lines derived from H4 cells. The oligonucleotides coding for α-synuclein shRNA, TRCNOOO320 and TRCN3736 (Sigma-Aldrich, St. Louis, USA), and scrambled shRNA were cloned into pLK0 harboring puromycine resistance. Lentiviruses were produced in HEK cells after transfection of shRNA plasmid, HIV-gag/pol (psPAX2, gift from Didier Trono, Addgene plasmid # 12260) and VSVG glycoprotein (pMD2, gift from Didier Trono, Addgene plasmid # 12259) using CalPhosMammalian transfection kit (Clontech, Mountain View, USA) according to the manufacturer’s instructions. 24 h after transfection, cells were washed with PBS and fresh DMEM with 10 % FCS was added. 48 h and 72 h post transfection, medium containing lentiviruses were collected and centrifuged at 300 g for 5 min to remove floating cells. H4 cells were infected using 80 % medium containing lentiviruses, 20 % DMEM with 10 % FCS and 6 μg/mL polybrene. After 24 h of incubation, cells were washed with PBS and stably transfected cells were selected with puromycine at a final concentration of 6 μg/ml.

### Immunhistochemistry of mouse brain tissue

Wt C57Bl/6 mice (1–3 months old, female) were anesthetized with ketamin/xylazin and perfused with PBS. After incubation of brains with 30 % sucrose for 24 h at 4 °C, brain tissue was embedded in TissueTek®O.C.T (Sakura, Alphen a.d.R., Nehterland), frozen at −80 °C and cut in 12 μm sections using a cryostat. Sections were fixed with 2 % PFA for 30 min, permeabilized with 0,5 % saponine for 10 min and treated with 3 % H_2_O_2_ for 10 min, followed by blocking with Roti®-ImmunoBlock (Roth, Karlsruhe, Germany) for 1 h. Samples were co-immunostained with primary antibodies mouse-anti-α-synuclein (1:200, syn-1,BD,New Jersey, USA) and rabbit-anti-SOD1 (1:100, ADI-SOD100, Enzo life science, New York, USA) in Roti®-ImmunoBlock for 1 h 45 min at RT. After washing with PBS, sections were blocked with 5 % goat-serum in PBS for 15 min and incubated with fluorophore conjugated secondary antibodies (1:750, goat-anti-rabbit-Alexa546 and goat-anti-mouse-Alexa-488, both Life technology, Carlsbad, USA) in 5 % goat serum for 1 h. Then sections were washed with PBS, incubated with xylol for 2 min and 100 % ethanol for 3 min and coverslipped using DAPI Fluoromont®G (SouthernBioTech, Birmingham, USA). To avoid unspecific binding of secondary antibodies, sections were also single stained with either α-synuclein or SOD1 primary antibody and both secondary antibodies.

### Immunhistochemistry of human brain sections

Immunohistochemistry of midbrain sections of a patient with DLB was performed as described previously [62]. In brief, 5 μm thick paraffin-embed human midbrain sections were de-paraffinized by xylol and a descending series of alcohols and subjected to antigen retrieval by steaming in dH_2_O for 30 min. Sections were blocked with 5 % goat serum in PBS-T for 1 h and incubated with primary antibodies mouse-anti- α-synuclein( 1:100, 4B12, Covance, Princeton, USA) and rabbit-anti-SOD1 (1:70, [60]) in 2,5 % goat serum at 4 °C overnight. After washing with PBS, sections were incubated with secondary antibodies (1:500, goat-anti-rabbit-Alexa568 and goat-anti-mouse-Alexa-488, both Life technology, Carlsbad, USA) in 1 % goat-serum for 1 h at RT. Subsequently sections were washed with PBS, blocked with 1 % Sudan Black (Sigma, St.Louis, USA) for 2 min and coverslipped with Fluoromont®G (SouthernBioTech, Birmingham, USA). As control, sections were stained without primary antibodies.

### Confocal and fluorescent microscopy

Confocal microscopy of mouse brain sections was performed using a Carl Zeiss LSM 710 laser scanning microscope (LSM 710 NLO, Carl Zeiss, Oberkochen, Germany) and a LD C-Apochromat 63x/1,15 W Korr objective. Confocal images were analyzed with the ZEN2010 software (Carl Zeiss Microimaging GmbH, Jena, Germany).

Immunostained mouse brain sections were also evaluated using a Carl Zeiss Axio Observer.A1 microscope and digital camera (AxioCamMRm, Zeiss, Oberkochen, Germany). Apart from same brightness and contrast correction for all images, no additional image processing was performed.

### Ethics, consent and permissions

Human blood sample collection was performed in accordance with the declaration of Helsinki and approved by the Ethic Committee of Ulm University. All volunteers gave informed written consent to participate in the study. Animal studies were performed in compliance with the National Institute of Health guidelines for the use of experimental animals.

### Quantification of western blots

Western blots were quantified with ImageJ (Version 1,48). Expression levels of proteins of interest were standardized to protein expression level of a loading control (e.g. β-actin). At least three western blots of three biological replicates/samples (n = 3) were quantified and used for statistical analysis.

### Statistical analysis

Graph Pad Prism (Version 5,04 and 6,05) was used to calculate the mean, standard error of mean (SEM) and to perform statistical analysis. The values are shown as mean ± SEM. Unless indicated otherwise, figures show pooled data from several independent replicates. To pool the data from independent experiments, the data of each experiment were normalized to the mean of the control and then used for statistical analysis. One way ANOVA/Tukey’s multiple comparison test and two tailed, unpaired student’s t-test were used to calculate the p-values. P values less than 0,05 were considered as significant.

## Additional files

Additional file 1: Figure S1. Concept of the extracellular complementation assay: Cells expressing proteins with n-terminal halve of hGluc or proteins with c-terminal halve of hGluc were lysed, followed by determination the concentrations α-synuclein and SOD1. Lysates were adjusted to the same molarity of α-synuclein or SOD1 and combined. After incubation, luciferase activity was measured. (TIF 2014 kb) Additional file 2: Figure S2. Luciferase signal of S1/SOD1-2 and S2/SOD1-1 is not based on a nonspecific binding of luciferase halves. (A) Luciferase activity measurement of living H4 cells and (B) conditioned medium 24 h post transfection with luciferase halves alone (L1 + L2) or luciferase halves tagged to α-synuclein and SOD1. Figure shows pooled data from 3 independent experiments after normalization to the respective mean of luciferase activity of co-transfected cells with L1 + L2 (two tailed, unpaired student’s t-test, *n* = 12, * *p* < 0,05, ** *p* < 0,005, *** *p* < 0,0005). (TIF 158 kb) Additional file 3: Figure S3. Co-IP studies confirm the α-synuclein-SOD1 binding. (A) α-Synuclein immunoprecipitation of co-transfected H4 cells using α-synuclein antibody co-immunoprecipitated SOD1-myc and endogenous SOD1. Input: 5 μg. (B) Immunoprecipitation using myc antibody co-immunoprecipitated α-synuclein. After α-synuclein detection, membrane was incubated with stripping buffer, blocked and incubated with an anti-tau antibody and secondary antibody. (C) α-Synuclein immunoprecipitation of wt mouse brain homogenate without the usage of the DSP crosslinking reagent detects co-immunoprecipitated SOD1. Input: 8 μg. (TIF 4670 kb) Additional file 4: Figure S4. Co-localization of α-synuclein and SOD1 in wt mouse brain. Representative images of C57Bl/6 wt mouse brain sections co-immunostained for α-synuclein (Alexa 488, green) and SOD1 (Alexa 546, red). Nuclei were stained with DAPI. As control, sections were stained either with α-synuclein or SOD1 primary antibody but with both fluorophores (Alexa 488, Alexa 546) conjugated secondary antibodies. (TIF 7729 kb) Additional file 5: Figure S5. Increase in luciferase activity in presence of α-synuclein and reduced luciferase activity in absence of α-synuclein does not derive from unequal expression levels. **A** Densitometry of western blots from H4 cell co-transfected with SOD1-1 /-2 complementation pair and with myc, wt, A30P or A53T α-synuclein. **B** Expression level of S1/S2 in the presence of myc, wt and mutated SOD1-myc relative to β-actin (two tailed, unpaired student’s t-test, *n* = 3). (C) Expression level of SOD1-1 and SOD1-2 of lysates from scrambled-shRNA stabile H4 cell line and two α-synuclein-shRNA stabile H4 cell lines (α-syn I, α-syn II) relative to β-actin (two tailed, unpaired student’s t-test, n = 4). (D) Densitometry of western blots from SOD1-1 and SOD1-2 co-transfected H4 cells treated with 7 μM recombinant α-synuclein or solvent PBS (two tailed, unpaired student’s t-test, n = 3). (n.s. = not significant). (TIF 964 kb)

## Abbreviations

PD: Parkinson’s disease
ALS: Amyotrophic lateral sclerosis
α-syn: Alpha-synuclein
SOD1: Super oxide dismutase 1
HGluc: Humanized *Gaussia* princeps luciferase
S1: α-syn fused to amino-terminal fragment of hGluc
S2: α-syn fused to carboxy-terminal fragment of hGluc
SOD1-1: SOD1 fused to amino-terminal fragment of hGluc
SOD1-2: SOD1 fused to carboxy-terminal fragment of hGluc
SOD1-3: Amino-terminal fragment of hGluc fused to amino-terminus of SOD1
SOD1-4: Carboxy-terminal fragment of hGluc fused to amino-terminus of SOD1
L1: Amino-fragment of hGluc
L2: Carboxy-fragment of hGluc
IHC: Immunhistochemistry
