# Supplementary figures and images for: α-synuclein interacts with SOD1 and promotes its oligomerization
Source: Mol Neurodegener. 2015 Dec 8;10:66. doi: 10.1186/s13024-015-0062-3 (PMC4672499; doi:10.1186/s13024-015-0062-3)

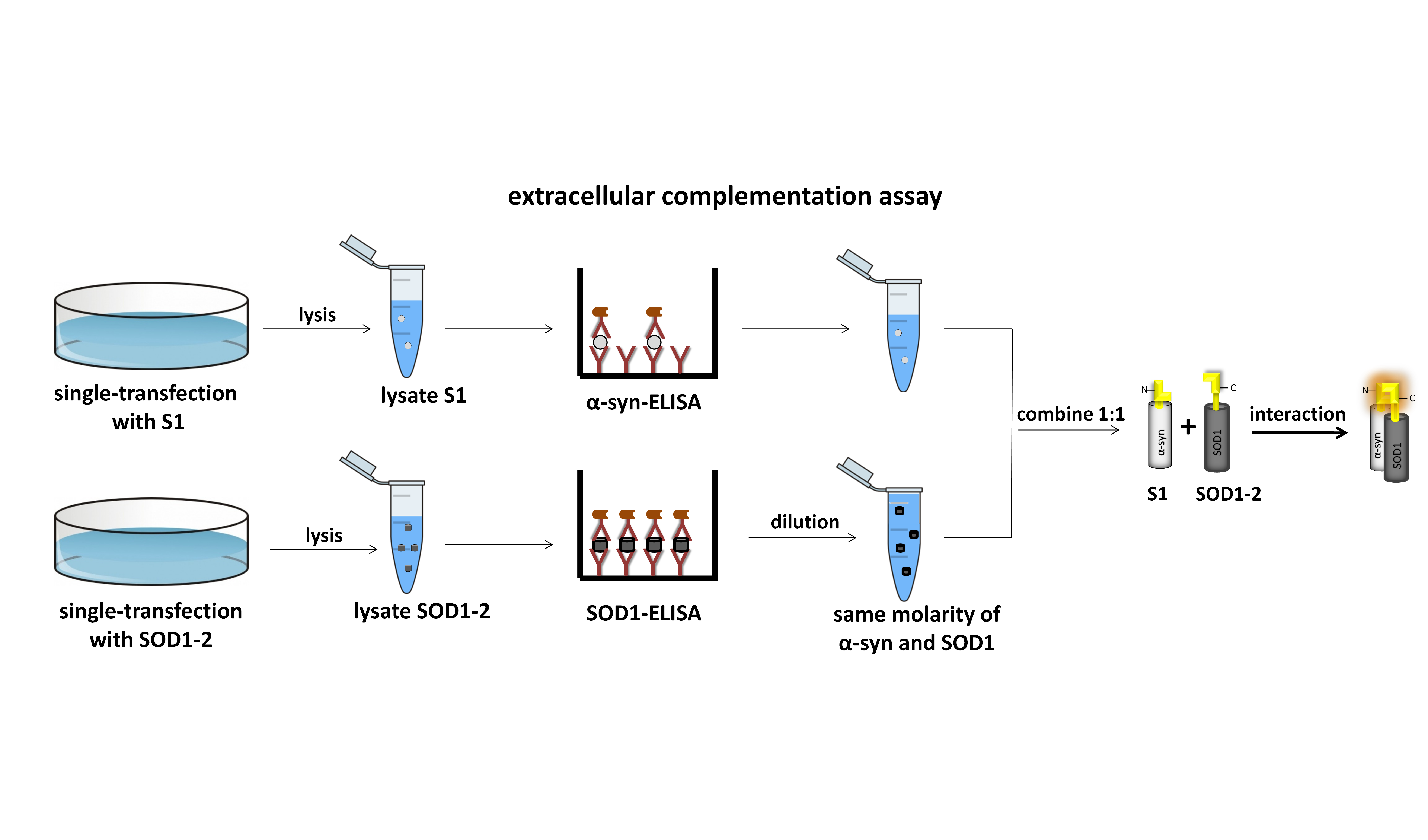

Supplement: Additional file 1: Figure S1. — Concept of the extracellular complementation assay: Cells expressing proteins with n-terminal halve of hGluc or proteins with c-terminal halve of hGluc were lysed, followed by determination the concentrations α-synuclein and SOD1. Lysates were adjusted to the same molarity of α-synuclein or SOD1 and combined. After incubation, luciferase activity was measured. (TIF 2014 kb) [file 13024_2015_62_MOESM1_ESM.tif]

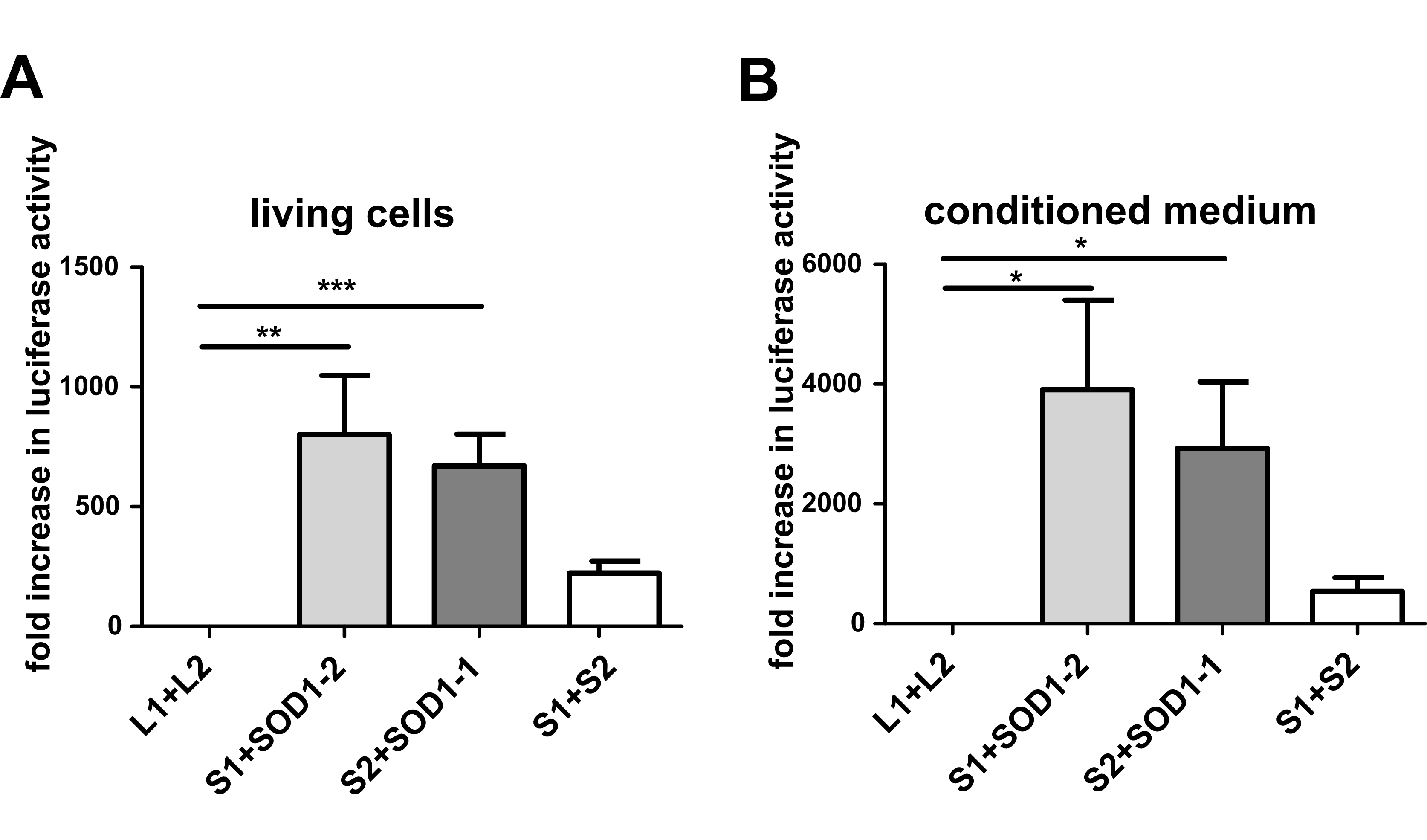

Supplement: Additional file 2: Figure S2. — Luciferase signal of S1/SOD1-2 and S2/SOD1-1 is not based on a nonspecific binding of luciferase halves. (A) Luciferase activity measurement of living H4 cells and (B) conditioned medium 24 h post transfection with luciferase halves alone (L1 + L2) or luciferase halves tagged to α-synuclein and SOD1. Figure shows pooled data from 3 independent experiments after normalization to the respective mean of luciferase activity of co-transfected cells with L1 + L2 (two tailed, unpaired student’s t-test, n = 12, * p < 0,05, ** p < 0,005, *** p < 0,0005). (TIF 158 kb) [file 13024_2015_62_MOESM2_ESM.tif]

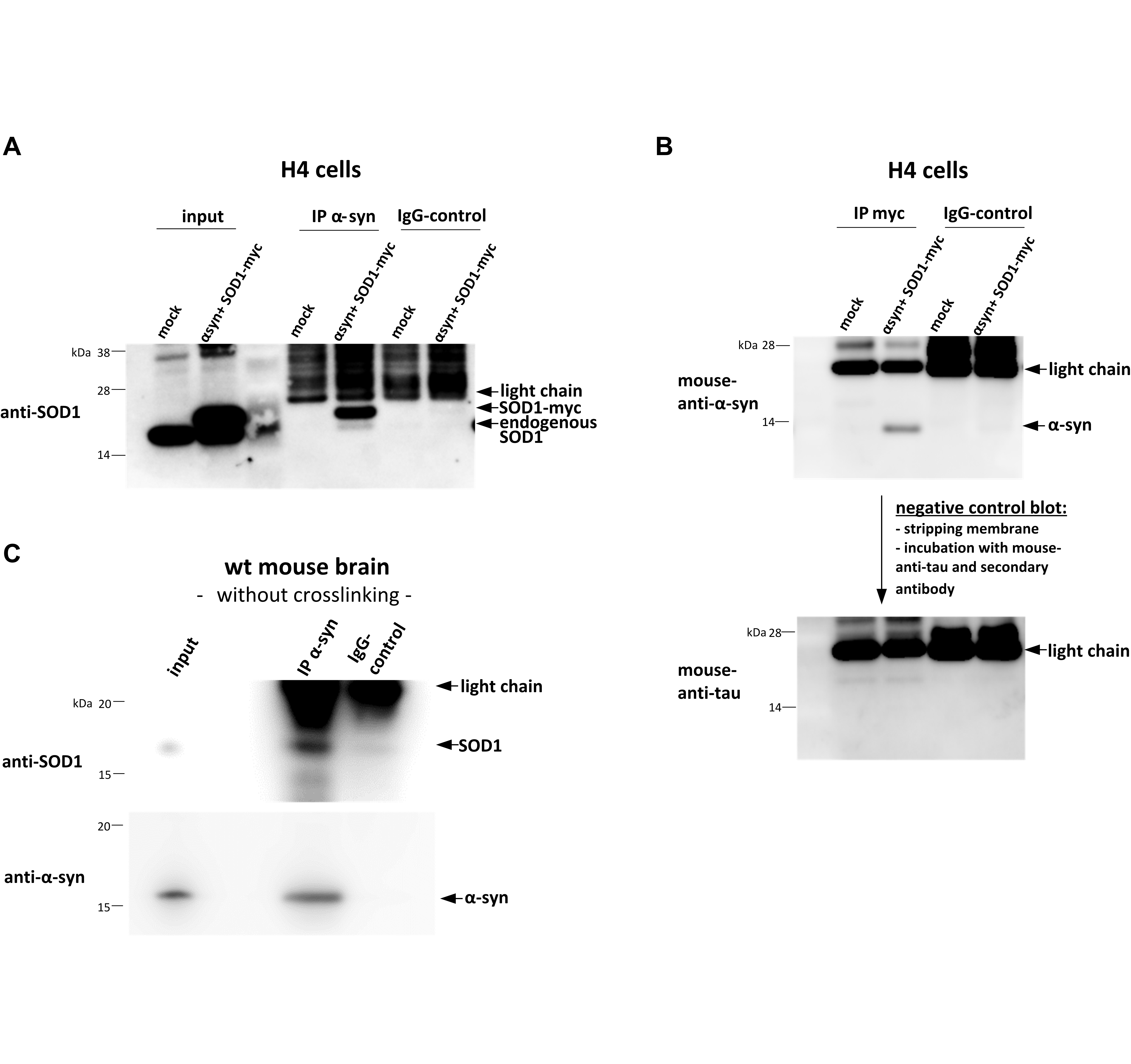

Supplement: Additional file 3: Figure S3. — Co-IP studies confirm the α-synuclein-SOD1 binding. (A) α-Synuclein immunoprecipitation of co-transfected H4 cells using α-synuclein antibody co-immunoprecipitated SOD1-myc and endogenous SOD1. Input: 5 μg. (B) Immunoprecipitation using myc antibody co-immunoprecipitated α-synuclein. After α-synuclein detection, membrane was incubated with stripping buffer, blocked and incubated with an anti-tau antibody and secondary antibody. (C) α-Synuclein immunoprecipitation of wt mouse brain homogenate without the usage of the DSP crosslinking reagent detects co-immunoprecipitated SOD1. Input: 8 μg. (TIF 4670 kb) [file 13024_2015_62_MOESM3_ESM.tif]

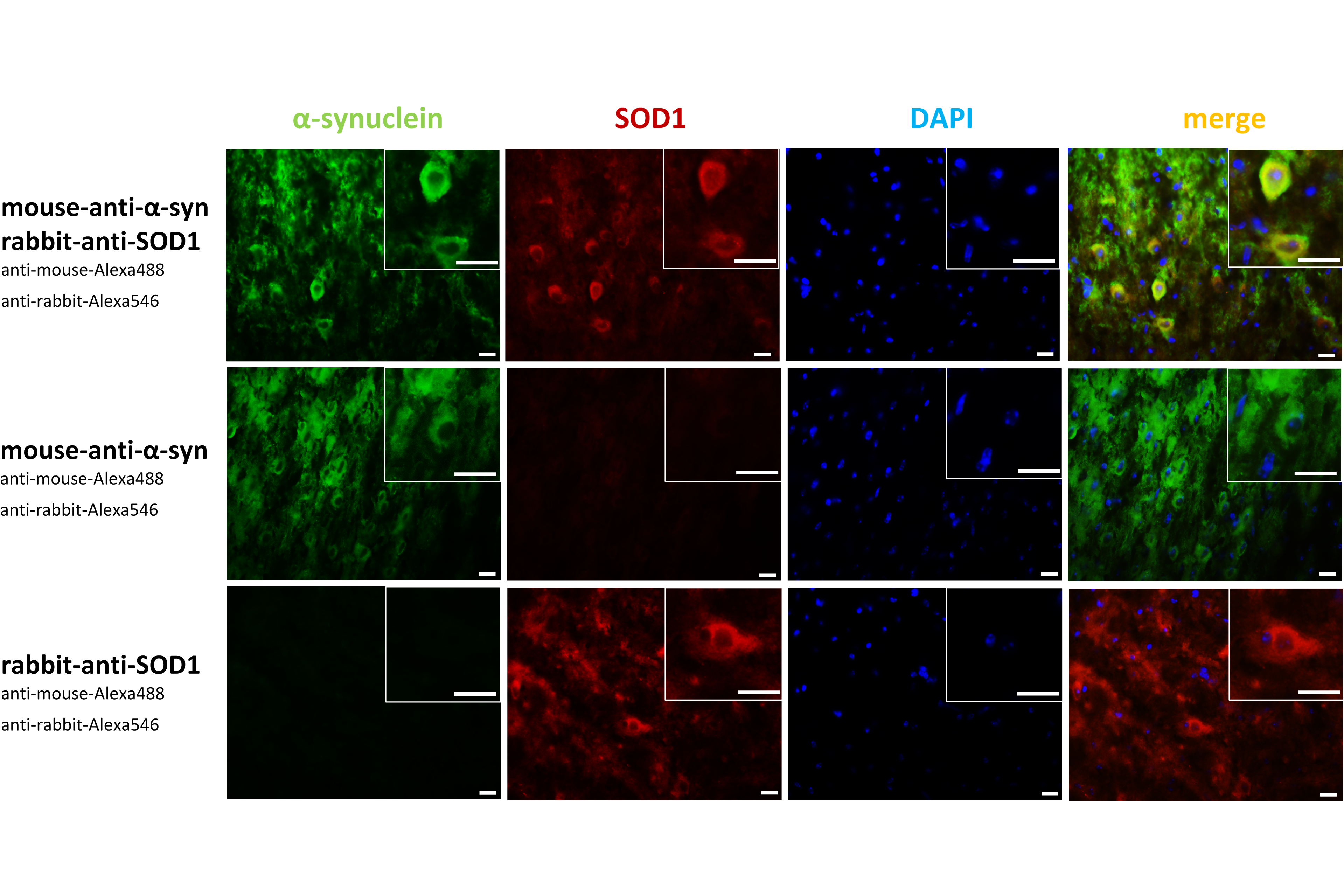

Supplement: Additional file 4: Figure S4. — Co-localization of α-synuclein and SOD1 in wt mouse brain. Representative images of C57Bl/6 wt mouse brain sections co-immunostained for α-synuclein (Alexa 488, green) and SOD1 (Alexa 546, red). Nuclei were stained with DAPI. As control, sections were stained either with α-synuclein or SOD1 primary antibody but with both fluorophores (Alexa 488, Alexa 546) conjugated secondary antibodies. (TIF 7729 kb) [file 13024_2015_62_MOESM4_ESM.tif]

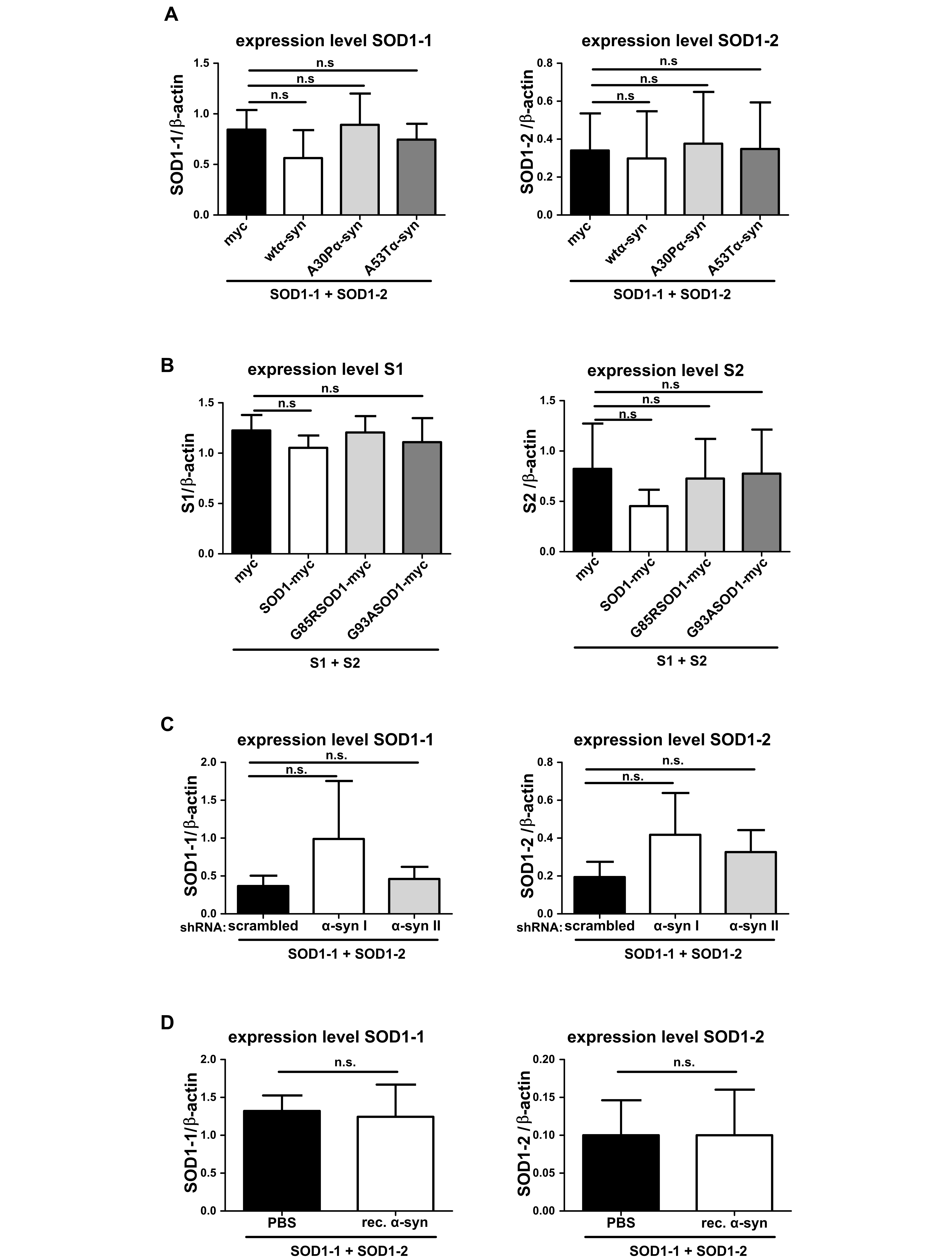

Supplement: Additional file 5: Figure S5. — Increase in luciferase activity in presence of α-synuclein and reduced luciferase activity in absence of α-synuclein does not derive from unequal expression levels. A Densitometry of western blots from H4 cell co-transfected with SOD1-1 /-2 complementation pair and with myc, wt, A30P or A53T α-synuclein. B Expression level of S1/S2 in the presence of myc, wt and mutated SOD1-myc relative to β-actin (two tailed, unpaired student’s t-test, n = 3). (C) Expression level of SOD1-1 and SOD1-2 of lysates from scrambled-shRNA stabile H4 cell line and two α-synuclein-shRNA stabile H4 cell lines (α-syn I, α-syn II) relative to β-actin (two tailed, unpaired student’s t-test, n = 4). (D) Densitometry of western blots from SOD1-1 and SOD1-2 co-transfected H4 cells treated with 7 μM recombinant α-synuclein or solvent PBS (two tailed, unpaired student’s t-test, n = 3). (n.s. = not significant). (TIF 964 kb) [file 13024_2015_62_MOESM5_ESM.tif]
